# Supplementary material for: Alleviating negative symptoms in schizophrenia using a virtual reality-based therapy targeting social reward learning (ENGAGE): Protocol for a randomised, controlled, assessor-blind pilot study
Source: PLoS One. 2025 Oct 8;20(10):e0331632. doi: 10.1371/journal.pone.0331632 (PMC12507218; doi:10.1371/journal.pone.0331632)
Supplement: S2 File — (PDF) [file pone.0331632.s002.pdf]

S2: Overview of modules in the ENGAGE VR-based therapy ENGAGE

Module 1: Introduction (sessions 1-2)

In VR, participants are asked to spontaneously react to the VR environment and register their emotional reaction (assessment, session 1). Participants then practice focusing their attention on positive elements in the virtual environment, supported by the therapist (session 2).

Module 2: Motivation (sessions 3-4)

In VR, participants practice raising their anticipatory pleasure (session 3), and activating and motivating an avatar to get started on a task (session 4).

Module 3: Social Reward (sessions 5-6)

In VR, participants practice sharing a positive memory with an avatar (session 5), and engaging in a pleasurable social activity with an avatar (session 6).

Module 4: Individually targeted (sessions 7-9)

This module can be used for continued repetition or elaboration of previous themes, or to work on issues maintaining the negative symptoms for the participant, such as rumination, self-criticism, paranoid thinking or lack of social skills.

Session 10

Wrap-up session used to evaluate the therapy goals, emphasise the participants' development, and revisit helpful transferable strategies learned in therapy.
