# Supplementary material for: Alleviating negative symptoms in schizophrenia using a virtual reality-based therapy targeting social reward learning (ENGAGE): Protocol for a randomised, controlled, assessor-blind pilot study
Source: PLoS One. 2025 Oct 8;20(10):e0331632. doi: 10.1371/journal.pone.0331632 (PMC12507218; doi:10.1371/journal.pone.0331632)
Supplement: S3 File — (PDF) [file pone.0331632.s003.pdf]

S3: Ecological Momentary Assessment (EMA) Protocol ENGAGE

Remarks on Operationalisation Choices

Consummatory anhedonia has previously been conceptualised as the relationship between positive affect and the occurrence of pleasant events (Fung et al., 2023; Hermans et al., 2021). Event pleasantness was determined by participants' ratings, leaving a risk of underestimating the true extent of anhedonia: Given that the phenomenon of anhedonia is likely to alter an individual's sensitivity to the positive emotional valence of events, anhedonic patients may report generally lower degrees of event pleasantness compared to healthy controls. These instances would then be omitted from further analysis, as associations with PA were tested for sufficiently pleasant-rated events only (Hermans et al., 2021). For the present study, therefore, anhedonia will be measured with a single item ("Hvor meget nyder du det du laver?" = "How much are you enjoying what you are doing?"), an operationalisation previously employed by Luther and colleagues (2023).

In addition to the assessment of positive emotional reaction, the present study will also capture a negative emotional response to activity and company. This approach allows for the investigation of *consummatory anhedonia* (Oorschot et al., 2013), but also recognises the possibility of negative emotional reactivity (Viinikainen et al., 2009). Assessing negative emotional response will enable the detection of event- or company-related *blunted affect*, which is considered a distinctive feature of negative symptoms in psychosis.

Some investigators have probed *anticipatory anhedonia* only in the presence of a specific consummatory experience (e.g., "How much do you think you will enjoy that activity the next time you do it?"; Luther et al., 2023), while others required participants to respond to several item iterations (e.g., "Which of the following activities do you anticipate doing within the next 2-3 hours?" – "How much do you think you will enjoy [X]?"; Moran et al., 2017). The present study will continue to assess anticipatory anhedonia at the occasion of a current activity, and adds an item assessing non-specific anticipatory pleasure ("Jeg glæder mig meget til noget i de næste 2-3 timer" = "I am much looking forward to something within the next 2-3 hours").

List of Items

Outcome		Item	Response	Logic
Positive affect (PA)[1]		[H] Lige nu føler jeg mig…<br>[L] I dag har jeg følt mig…		
	1	glad	0–100[2]	
	2	begejstret	0–100	
	3	afslappet	0–100	
	4	tilfreds	0–100	
Negative affect (NA)		[H] Lige nu føler jeg mig…<br>[L] I dag har jeg følt mig…		
	5	ængstelig	0–100	
	6	nedtrykt	0–100	

[1] Items 1–8: cf. Hermans et al. (2021)
[2] Likert scale as a visual slider

[H] High-frequency epochs E1 and E3
[L] Low-frequency epoch E2

	7	nervøs	0–100	
	8	irriteret	0–100	
Activity (A)[3]	9	[H] Hvad laver du lige nu?<br>[L] Hvad har du lavet i dag?	☐ Arbejde/studere/lære[4]	+11-13
			☐ Spise/drikke	+11-13
			☐ Lave mad	+11-13
			☐ Hobby/hygge	+11-13
			☐ Internet/computer	+11-13
			☐ Gå ærinder/husarbejde	+11-13
			☐ Slappe af	+10
			☐ Træne	+11-13
			☐ Handle/shoppe	+11-13
			☐ Fjernsyn/musik	+11-13
			☐ Rejse	+11-13
			☐ Være ude i naturen	+11-13
			☐ Ryge	+11-13
			☐ Brusebad/karbad	+11-13
			☐ Gå hvileløst rundt	+11-13
			☐ Tilbringe tid sammen med nogen	+11-13
			☐ Andet	+freetext<br>+11-13
			☐ Intet[5]	+10
Avolition (AV)	10	[H] Hvor meget ville du ønske, at du lavede noget lige nu?<br>[L] Hvor meget ville du ønske, at du havde lavet noget i dag?	0–100	*see nr. 9*
Consummatory anhedonia (cAH) Blunted affect (BA)	11	[H] Jeg nyder fuldt ud det jeg laver lige nu.<br>[L] Jeg har fuldt ud nydt mine aktiviteter i dag.	0–100	*see nr. 9*
Blunted affect (BA)	12	[H] Jeg synes slet ikke om det jeg laver lige nu.<br>[L] Jeg syntes slet ikke om mine aktiviteter i dag.	0–100	*see nr. 9*
Avolition (AV)	13	[H] Hvor motiveret er du til din aktuelle aktivitet?<br>[L] Hvor motiveret har du været til dine aktiviteter i dag?	0–100	*see nr. 9*
Company (C)[6]	14	[H] Hvem er du sammen med lige nu? *Dette gælder både nar I er fysisk til stede sammen, taler i telefon,*	☐ Kæreste, partner, ægtefælle e.l.	+16-18
			☐ Familie eller sambo(er)	+16-18
			☐ Ven(ner)	+16-18

[3] Items 9–10, 13: based on Raugh et al. (2020)
[4] ☐ = Multiple Selection
[5] Cannot be selected together with the other options
[6] Items 14–15, 18: based on Raugh et al. (2020)

		videosamtaler eller på skrift (fx sms eller chats).	☐ Kolleg(er) / klassekammerater	+16-18
			☐ Læge / terapeut	+16-18
			☐ Fremmede	+16-18
			☐ Anden	+freetext +16-18
			☐ Jeg er alene[7]	+15
		[L] Hvor mange mennesker har du været sammen med i dag?	☐ (fritekst)	+16-18
			☐ Jeg var alene[8]	+15
	15	[H] Hvor meget ville du ønske, at du var sammen med nogen lige nu?<br>[L] Hvor meget ville du ønske, at du havde været sammen med nogen i dag?	0–100	*see nr. 14*
Asociality (AS) Blunted affect (BA)	16	[H] Jeg nyder fuldt ud at være sammen med vedkommende.<br>[L] Jeg har fuldt ud nydt at være sammen med de mennesker, jeg var sammen med i dag.	0–100	*see nr. 14*
Blunted affect (BA)	17	[H] Jeg synes slet ikke om at være sammen med vedkommende.<br>[L] Jeg har slet ikke syntes om at være sammen med de mennesker, jeg var sammen med i dag.	0–100	*see nr. 14*
Asociality (AS)	18	[H] Hvor motiveret er du til at være sammen med vedkommende?<br>[L] Hvor motiveret har du været til at være sammen med andre mennesker i dag?	0–100	*see nr. 14*
Anticipatory anhedonia (aAH)	19	[H] Jeg glæder mig meget til noget i de næste 2-3 timer.<br>[L] Jeg glæder mig meget til noget i morgen.	0–100	

[7] Cannot be selected together with the other options
[8] Cannot be selected together with the other options

References

Fung, V. S. C., Chan, J. C. Y., Wong, S. C. Y., Wong, C. S. M., Kirtley, O., Myin-Germeys, I., Strauss, G. P., & Chang, W. C. (2023). Investigation of momentary negative symptoms in patients with early psychosis in daily life: An experience sampling study. *Psychiatry Research*, *325*, 115234. https://doi.org/10.1016/j.psychres.2023.115234

Hermans, K. S. F. M., Myin-Germeys, I., Gayer-Anderson, C., Kempton, M. J., Valmaggia, L., McGuire, P., Murray, R. M., Garety, P., Wykes, T., Morgan, C., Kasanova, Z., & Reininghaus, U. (2021). Elucidating negative symptoms in the daily life of individuals in the early stages of psychosis. *Psychological Medicine*, *51*(15), 2599–2609. https://doi.org/10.1017/S0033291720001154

Luther, L., Raugh, I. M., Collins, D. E., Knippenberg, A. R., & Strauss, G. P. (2023). Negative symptoms in schizophrenia differ across environmental contexts in daily life. *Journal of Psychiatric Research*, *161*, 10–18. https://doi.org/10.1016/j.jpsychires.2023.02.037

Moran, E. K., Culbreth, A. J., & Barch, D. M. (2017). Ecological momentary assessment of negative symptoms in schizophrenia: Relationships to effort-based decision making and reinforcement learning. *Journal of Abnormal Psychology*, *126*(1), 96–105. https://doi.org/10.1037/abn0000240

Oorschot, M., Lataster, T., Thewissen, V., Lardinois, M., Wichers, M., van Os, J., Delespaul, P., & Myin-Germeys, I. (2013). Emotional Experience in Negative Symptoms of Schizophrenia—No Evidence for a Generalized Hedonic Deficit. *Schizophrenia Bulletin*, *39*(1), 217–225. https://doi.org/10.1093/schbul/sbr137

Raugh, I. M., James, S. H., Gonzalez, C. M., Chapman, H. C., Cohen, A. S., Kirkpatrick, B., & Strauss, G. P. (2020). Geolocation as a Digital Phenotyping Measure of Negative Symptoms and Functional Outcome. *Schizophrenia Bulletin*, *46*(6), 1596–1607. https://doi.org/10.1093/schbul/sbaa121

Viinikainen, M., Jääskeläinen, I. P., Alexandrov, Y., Balk, M. H., Autti, T., & Sams, M. (2009). Nonlinear relationship between emotional valence and brain activity: Evidence of separate negative and positive valence dimensions. *Human Brain Mapping*, *31*(7), 1030–1040. https://doi.org/10.1002/hbm.20915

[H] High-frequency epochs E1 and E3
[L] Low-frequency epoch E2
