# Supplementary material for: Alleviating negative symptoms in schizophrenia using a virtual reality-based therapy targeting social reward learning (ENGAGE): Protocol for a randomised, controlled, assessor-blind pilot study
Source: PLoS One. 2025 Oct 8;20(10):e0331632. doi: 10.1371/journal.pone.0331632 (PMC12507218; doi:10.1371/journal.pone.0331632)
Supplement: S4 File — (PDF) [file pone.0331632.s004.pdf]

ENGAGE pilot study: Alleviating negative symptoms in schizophrenia using a virtual reality-based intervention targeting social reward learning

ENGAGE pilotstudie: reduktion af negative symptomer ved skizofreni ved brug af en virtual-reality baseret intervention rettet mod social belønning

Forsøgsprotokol

Version 2.2

Forsøgsleder Louise Birkedal Glenthøj, dr.med, PhD, specialpsykolog, specialist i psykoterapi, lektor og forskningsleder VIRTU Research Group, Psykiatrisk Center København, Københavns Universitet

Indhold

1. Aim ... 3
2. Background ... 3
3. Hypotheses ... 4
4. Method ... 4
5. Virtual reality-based intervention ... 4
6. Adverse events ... 4
7. Outcomes ... 5
8. Randomisation and blinding ... 5
9. Statistical analyses and sample size calculation ... 5
10. Organization ... 6
11. Feasibility ... 6
12. Clinical impact ... 6
13. Dissemination ... 7
14. Research plan ... 7
15. Dansk tillæg til forsøgsprotokol ... 8
16. References ... 15

ENGAGE pilot study: Alleviating negative symptoms in schizophrenia using a virtual reality-based intervention targeting social reward learning

1. Aim

The ENGAGE pilot study targets negative symptoms (NS) being a critical unmet treatment need as identified by clinicians and in the literature(Correll & Schooler, 2020) using an innovative intervention that can be scaled for clinical implementation. The pilot study aims to provide essential knowledge on the feasibility, acceptability, and preliminary efficacy of a short-term, targeted, VR-based treatment expected to alleviate NS, improve daily life functioning and quality of life, along with potentially altering the reward processing system in patients with schizophrenia.

2. Background

Schizophrenia is a severe mental disorder affecting young people in their adolescence or early adult life and can have lifelong implications for employment, relationship etc.(Global Burden of Disease Study 2013 Collaborators, 2015; Hakulinen et al., 2019; Hjorthøj et al., 2017). Negative symptoms (NS) are core features of schizophrenia and strongly correlated with vocational and social outcomes(Ventura et al., 2009), quality of life(Eack & Newhill, 2007), and have a higher impact on later outcome than positive symptoms(Albert et al., 2011; Austin et al., 2015). While there are good medical treatment options for psychotic symptoms, no robust and replicated evidence exists for effective NS treatments(Fusar-Poli et al., 2015; Galderisi et al., 2021). Consistent evidence suggests that abnormalities of the brain reward system are central in the pathogenesis of NS, i.e., the domains of anhedonia and avolition(Gold et al., 2008; Nielsen et al., 2018; Strauss et al., 2014). Preliminary evidence indicates changes in reward learning following therapy is related to a reduction in NS(Cella et al., 2014).
Virtual reality (VR) is a promising technologically advanced mental health research tool with high ecological validity(Freeman et al., 2017). VR allows individuals to immerse themselves in realistic environments and interact with virtual objects, providing a safe and controlled space to address symptoms and improve functioning. Initial evidence (N=4) supports that VR-based interventions significantly reduce NS and improve psychosocial functioning with a medium to large effect size (0.6)(Novo et al., 2021). Given the suggested central role of reward processing disturbances in the pathogenesis of NS, there is an obvious need to investigate the effect of a VR-based intervention targeting reward processing in ameliorating NS. Both NS and motivational abnormalities reflect complex processes at a system level likely corresponding to functional brain activity within and between several regions and circuits(Bègue et al., 2020). Thus, a task-based approach investigating functional responses to different types of reward appears most beneficial in elucidating associated neurobiological processes(Wang et al., 2016). The identification of specific connectivity features associated with the distinct psychopathological factors is essential when aiming to elucidate the potential for remediating abnormal reward processing(Van Der Meer et al., 2021). Supplementing the clinical assessments in the study with imaging data may clarify whether the intervention directly targeting reward processing may have any effect on the biological processes/networks involved.
In sum, this study will explore the prospects of using an innovative, VR-based treatment to treat NS and reward processing deficits in schizophrenia aimed at producing real-life benefits for the patients. Supplementing comprehensive clinical outcome evaluation, imaging data will elucidate the neuroplastic potential of the intervention which will move the field of clinical psychiatry forward and is an important step toward refining and personalizing treatment approaches.

3. Hypotheses

In patients with schizophrenia: 1) VR-based therapy along with the study procedures will be feasible and acceptable 2) VR-based therapy will indicate improvements in reward processing, decrease NS, and improve functioning and quality of life.

4. Method

The study will enroll 30 patients with schizophrenia from the psychiatric outpatient clinics in the Capital Region of Denmark. Participants will be randomized to either 10 sessions of a VR-based intervention or treatment as usual. Patients will be assessed at baseline and at treatment cessation (3-months). Additionally, five healthy controls (HC) will be recruited to complete a single MRI scan session to serve as a reference cohort. The study will be approved by the Danish Health Research Ethic committee.

4.1. Participants

Inclusion criteria: 18 – 70 years, ICD-10 diagnosis of a psychotic disorder (F2x), negative symptom score of ≥3 on the SANS items avolition or anhedonia. Exclusion criteria: diagnosis of organic brain disease, Intellectual disability (IQ < 70), current diagnosis of drug dependency, a command of spoken Danish or English inadequate for engaging in therapy, refuse being informed on incidental findings on the MRI-scans. The HC comparison group will meet the same criteria, absent clinical diagnosis.

5. Virtual reality-based intervention

The manualized intervention comprises 10 individual sessions addressing the core NS of anhedonia and avolition that are linked to the social reward processing system. Therapy length is in line with positive findings of short-term interventions targeting reward processing in psychiatric disorders[27,28] along with review findings of ≥8 sessions of non-pharmacological interventions being required to achieve neuroplastic changes[29]. The VR-therapy aims at enhancing anticipatory pleasure and positive emotions via a virtual exposure to social environments using the highly elaborate *Social World* treatment program. The VR exposure comprises eight virtual social environments (e.g., a bus, café, etc.). The program allows for an individualized and tailored treatment approach. By engaging the participant in virtual situations, the therapy aims to amplify the exposure and reactivity towards pleasurable and engaging events. The therapy focuses on increasing the awareness of positive outcomes that may influence motivation. Finally, the therapy deploys activity appraisal/re-appraisal intended to enhance the salience of future opportunities for reward and desire for such rewards, thus targeting reward anticipation processes and promoting enjoyable daily-life activities. The VR-program is proven well-tolerated and effective in psychotic disorders(Pot-kolder et al., 2018).
All therapy sessions in the experimental group will be audiotaped. A selected number of sessions will be rated by an independent rater to ensure fidelity to the treatment protocol.

6. Adverse events

Virtual reality therapy is generally well tolerated and with minimal or none side effects or adverse events(Rus-Calafell et al., 2018). There are, though, few reports of cyber sickness caused by the virtual reality therapy(Pot-kolder et al., 2018). Side effects and adverse events will be monitored and recorded throughout the study period.

7. Outcomes

7.1. Feasibility and acceptability

Feasibility and acceptability will be evaluated as recruitment of ≥80% of the target sample in 15 months, 70% retention to study protocol at cessation of therapy (10 sessions) and 80% reporting a satisfaction rating of ≥7 on a Likert scale. Qualitative interviews will assess participants' experience with the VR-treatment.

7.2. Clinical assessments

Scale for the Assessment of Negative Symptoms(Andreasen, 1984), Brief Negative Symptom Scale(Kirkpatrick et al., 2011), Self-evaluation of Negative Symptoms(Dollfus et al., 2016), Anticipatory and Consummatory Interpersonal Pleasure Scale(Gooding & Pflum, 2014), Scale for the assessment of positive symptoms (SAPS), Natural Language Processing (NLP), Personal and Social Performance scale(Morosini et al., 2000), Social Functioning Scale(Birchwood et al., 1990), WHO-5(Newnham et al., 2010), Defeatist Performance Attitude Scale(Grant & Beck, 2009), Calgary Depression Scale(Addington et al., 1993), Emotion Recognition Task(Sahakian & Owen, 1992), Prescence questionnaire(Witmer BG, 1998), Simulator Sickness Questionnaire(Kennedy et al., 1992), Readiness for Therapy Questionnaire(Ghomi et al., 2021), Client Satisfaction Questionnaire(Attkisson & Zwick, 1982). Additionally, ecological momentary assessment (EMA) will be employed that allows for capturing momentary features of negative symptoms (e.g. time spent home/alone, sedentary behavior). EMA is an advance in NS assessment and has effectively been used in previous negative symptom intervention studies(Browne et al., 2022).

7.3. Imaging

MRI scans are acquired with a Philips 3.0 T whole body MRI scanner with a 32-channel SENSE head coil. The task-based fMRI paradigm Social Incentive Delay (SID) task[39] will be used to measure social reward anticipation. Complementary to the SID, participants will complete a version of the monetary incentive delay task, which has revealed differences in reward processing in patients with schizophrenia and serves as a useful benchmark for establishing the relative strengths of social reward processing. Additionally, resting state fMRI will be acquired. Key regions of interest for the study include comprise mesocorticolimbic and fronto-striatal circuits including ventral tegmental area, amygdala, hippocampus, striatum, nucleus accumbens, and prefrontal cortex(Abram et al., 2017; Forlim et al., 2020; Giordano et al., 2022; Grill et al., 2021; Spreckelmeyer et al., 2009). MRI scans can result in incidental findings. In that case, a neuroradiological description of the MR images will be obtained from Rikke Norling MD, neuroradiologist at Rigshospitalet. If the neuroradiologist observes any suspect changes, participants and their general practitioner will be informed of the findings to decide on potential further assessment. Prof. and consultant Bjørn Ebdrup will be clinically responsible for MRI-scans.

8. Randomisation and blinding

Randomisation will be centralised and computerised with a concealed randomisation. Block size will be unknown to the investigators and clinicians. The randomised intervention allocation is concealed until the statistical analyses of resulting data have been completed. Assessors engaged in outcome evaluation are blinded.

9. Statistical analyses and sample size calculation

A mixed-methods design will be used. ANOVAs will evaluate the differences in clinical measures. Feasibility of trial procedures will be examined using proportions and exact Clopper Pearson's 95% confidence

intervals for assessments of feasibility and acceptability in terms of recruitment and treatment retention. The sample size of 30 participants is considered adequate for a pilot-study assessing feasibility, acceptability, and providing indications of intervention efficacy(Bell et al., 2018).

10. Organization

The clinical assessments and intervention will be conducted at VIRTU Research Group (headed by Louise B Glenthøj, LBG), and the MRI-scans at Functional Imaging Unit, Glostrup Hospital in collaboration with prof. Bjørn Ebdrup and assoc. prof. Patrick Fisher, Rigshospitalet, an expert in MRI-studies evaluating reward-related processing in clinical cohorts. The study has collaboration from assoc. prof. Gregory Strauss, University of Georgia, an international expert on the assessment and treatment of negative symptoms in schizophrenia (support letter enclosed). Additionally, there is an established collaboration with clinical heads of early psychosis facilities (OPUS) in Copenhagen (Rikke Hilker PhD and Marianne Melau PhD) that have provided clinical expertise in developing the study design and intervention targets (collaboration letter enclosed). A panel of people with lived experiences from OPUS will give feedback on the intervention in the study aiming at increasing therapy effectiveness and tolerability. A research assistant (RA) will conduct recruitment, clinical assessment, qualitative interviews, along with data analyses and scientific reporting of the study findings supervised by LBG that will be responsible for the scientific products as study PI. LBG continuously receives unsolicited applications from psychologists or MD's and the recruitment of an RA is therefore expected to be unproblematic.

11. Feasibility

LBG heading VIRTU Research Group has great proficiency in conducting VR-based treatments in psychiatry through eight large-scale ongoing trials comprising the psychosis population e.g(Jeppesen et al., 2022; Smith et al., 2022). The intervention to be tested is manualized and will be delivered by therapist Lise Mariegaard, specialized in VR-based therapies in psychosis. VIRTU has great expertise in completing all clinical/administrative documents needed for clinical trials along with proven capability of handling large data set and perform planned analyses. OPUS teams in Copenhagen enroll 500 new patients each year. Based on data from the OPUS cohort(Albert et al., 2017), we know that 49% of OPUS patients nationwide suffer clinical relevant levels of NS (SANS score on anhedonia/avolition of ≥3) and it is therefore highly realistic to include the target number of 30 participants within the 15 months study period.

12. Clinical impact

The study specifically addresses the DFF thematic call and aligns with the 10-year plan for psychiatry, which have a key focus on enhancing treatment options for severe mental disorders. Additionally, the study places significant emphasis on technological innovation by utilizing a highly promising VR-based intervention. Furthermore, it is conducted through a strong partnership involving service-users, clinicians, and esteemed national and international researchers.
If the pilot study is successful, it serves as the foundation for testing the intervention in an appropriately powered efficacy randomized clinical trial that may also include cost-effectiveness analyses of the intervention. As review findings support a transdiagnostic approach to anhedonia and avolition and social reward processing abnormalities(Whitton et al., 2015), the intervention can easily be adapted for use in other relevant target groups comprising depression, bipolar disorder(Zald & Treadway, 2017), or individuals at ultra- high risk for psychosis(Devoe et al., 2020), where it may serve a preventive purpose.
Supplementing the clinical outcome evaluation with neuroimaging-based will aid in clarifying the mechanisms by which the VR-based intervention targeting reward processing networks may influence the brain's functional and structural architecture potentially indicating more durable effects of the intervention

along with aiding in delivering a personalized therapy. Overall, the study will be an important step towards developing more effective interventions for patients suffering from schizophrenia and serve to improve their clinical and functional prognosis.

13. Dissemination

Study results will be disseminated at relevant conferences, in scientific journals, through NGO's etc. Tentative papers: 1) Targeting reward related negative symptoms in schizophrenia using a VR-based intervention: Study protocol for the ENGAGE study. 2) Elucidating clinical, daily life, and neural changes following a VR-intervention aimed at alleviating negative symptoms 3) Baseline associations between aspects of negative symptoms and reward abnormalities in schizophrenia.

14. Research plan

	2024				2025				2026			
Approvals: Data Protection and Ethical Committee. REDCap database		x										
Feedback on therapy from service-users/clinicians; finalizing intervention		x	x									
EMA-assessment set-up + finalizing MRI-paradigm		x	x									
Study initiation: Recruitment and 3-months follow-up				x	x	x	x	x				
Analyses, writing of manuscripts									x	x	x	

For an English translation of the Danish language addendum, see pp. 20ff.

15. Dansk tillæg til forsøgsprotokol

15.1. Informeret samtykke og rekruttering

Rekruttering af patienter vil ske i samarbejde med sundhedspersonale fra de psykiatriske enheder i Region Hovedstadens Psykiatri eller i Region Sjælland. Personalet ambulante behandlingsenheder formidler kontakten til de projektansvarlige, såfremt patienten indvilliger heri.

Projektet vil også blive annonceret på Region Hovedstadens Psykiatris egen side, Forskningsportal Til Patientinklusion. Denne platform er specifikt designet til at præsentere igangværende forskningsprojekter for patienter og deres pårørende, som kunne være interesserede i at deltage i sådanne studier.

Uanset rekrutteringsform er det en forudsætning, at patienten opfylder kriterierne for deltagelse i projektet, og at patientens primære behandler bliver informeret før projektstart.

Der fremsendes/overleveres skriftlig information om projektet minimum 48 timer før første fremmøde i VIRTU Research Group, Psykiatrisk Center København, og der informeres om retten til at medbringe en bisidder.

Under forløbet vil de forsøgsansvarlige have kontakt med patientens kontaktperson i f.eks. OPUS eller distriktspsykiatrisk center, såfremt patienten samtykker til dette. Det er en forudsætning, at patienten opfylder kriterierne for deltagelse i projektet før projektstart.

Ved fremmødet informeres patienten mundtlig om projektet af projektets forskningsassistent og denne svarer på eventuelle spørgsmål før patienten underskriver samtykke erklæringerne. Patienten tilbydes en betænkningstid på 24 timer efter at have modtaget mundtlig og skriftlig information om projektet. Patienter, der selv udtrykker ønske herom, kan samtykke med det samme. Pjecen ”Forsøgspersoners rettigheder i sundhedsvidenskabelige forskningsprojekter” udleveres sammen med deltagerinformationen.

Samtalen vil foregå i et aflukket lokale eventuelt sammen med patientens bisidder. I tilfælde af spørgsmål eller komplikationer vil patienten i hele forsøgsperioden have mulighed for telefonisk at kontakte de forsøgsansvarlige eller andre sundhedsfaglige personer tilknyttet VIRTU Research Group, Psykiatrisk Center København.

Under forløbet vil de forsøgsansvarlige have kontakt med patientens kontaktperson i psykiatrisk behandlingstilbud, såfremt patienten samtykker til dette.

15.2. Kontakthyppighed

Ved baseline samt 3 måneder efter behandlingsafslutning foretages assessment til vurdering af psykopatologi, funktionsniveau MR-skanning samt bivirkninger/negative hændelser. Hvis en forsøgsperson afbryder protokollen, vil der blive tilbudt behandling efter relevante guidelines i psykiatrien i Region Hovedstaden eller Region Sjælland.

Kriterier for diskontinuation

Forsøgsdeltagerne kan når som helt forlade studiet, det er frivilligt at deltage. Hermed specificeret årsager til afbrydelse af studiet:

i. Frivillig afbrydelse af patienten selv; Patienten kan når som helst afbryde deltagelsen i forsøget uden at dette vil påvirke dennes videre behandling i sundhedssystemet.
ii. Forkert inklusion af patienten (f.eks. at patienten ikke opfylder inklusions/eksklusionskritererne).

Procedure for discontinuation

Patienter, som afbryder interventionen under indlæggelse, er fuldt berettiget til dette (jf. frivillighedsprincippet). Patienter, der ønsker at trække sig fra interventionen, vil blive undersøgt forelagt muligheden for at svare på spørgeskema omkring deres tilfredshed med behandlingen, hvis vedkommende ønsker dette.

15.3. Undersøgelser i studiet

Symptom- og funktionsniveau

Som anført i den engelske forsøgsprotokol, udføres assessments til vurdering af patienternes symptomniveau og funktionsniveau ved hjælp af interviews og selvrapporteringsskemaer. Yderligere stilles der mere kvalitative spørgsmål til deltagerne. De kvalitative spørgsmål omhandler deltagernes oplevelse af det at modtage terapi ved brug af virtual reality og hvad de oplevede var særligt effektivt eller kunne forbedres.

Fri tale interview

Deltagerne vil gennemgå et fri tale-interview med en forskningsassistent af 15-20 minutters varighed. Dette optages på lydoptager og omhandler spørgsmål om begivenheder i deltagerens liv og vedkommendes mentale helbred.

15.4. Digital assessment

Deltagerne vil yderligere modtage spørgsmål til mobiltelefon omkring deres symptomniveau og funktionsniveau. Dette via appen Monsenso. Denne kan downloades til egen telefon eller der kan udleveres smartphone til deltagerne i projektperioden, afhængig af, hvad deltageren foretrækker. Appen registrerer i tillæg geolokations- og accelerationsdata, der vil bruges for at kortlægge brugernes aktivitetsniveau. Formålet med den digitale assessment er at få et mere økologisk validt mål for fluktuationer i symptom- og funktionsniveau og herved mere detaljeret kunne beskrive mulige ændringer som følge af interventionen. I ugen op til eventuel start af intervention samt ugen umiddelbart efter afslutning af interventionen sendes

spørgsmål fem gange dagligt via appen med det formål at få et detaljeret billede af deltagernes hverdags funktions- og symptomniveau. I den øvrige 12-ugers projektperiode sendes spørgsmål en gang dagligt (aften).

15.5. MR-skanning

Scanningerne er beskrevet i den engelske forsøgsprotokol ovenfor. Med MR-skanning gives mulighed for at få strukturelle og funktionelle data om hjernens funktion. Scanningerne foretages af trænet personale på Glostrup Hospital og varer maksimalt 90 minutter inkl. pause. Professor og overlæge Bjørn Ebdrup vil være klinisk ansvarlig for MR-skanninger.

15.6. Tidsforbrug

Samlet tager undersøgelserne ca. 4 timer at gennemføre ved baseline og ca. 3 timer ved 3 måneders opfølgning.

15.7. Behandlingsmodaliteter

Den manualiserede, virtual reality intervention er beskrevet i den engelske forsøgsprotokol.

Medicinering

Der tilbydes ikke medikamentel behandling som del af dette studium. Patienternes eventuelle psykofarmakologiske behandling varetages af patientens primærbehandler i deres psykiatriske tilbud. Såfremt en projektdeltager er i medikamentel behandling, og ved kontakt med projektet oplyser om bivirkninger hertil, vil man i forskningsprojektet rette henvendelse til patientens primærbehandler.

Forsøgets bivirkninger, risici og ulemper

Der kan være bivirkninger ved forsøget i form af ubehag ved at bruge virtual reality briller og hovedtelefoner og eventuelt oplevelse af at blive rundtosset eller køresyg, men generelt rapporteres der ikke bivirkninger til virtuel reality behandling[40]. Den afprøvede form for terapi, der er sammenlignelig med VR-terapi, som vi har afprøvet i andet forsøg til reduktion af paranoia(Jeppesen et al., 2022), og som yderligere har været afprøvet internationalt(Pot-kolder et al., 2018), har udelukkende vist gavnlig effekt i forhold til at reducere patienternes symptomniveau og deres forpinthed og ubehag forbundet hermed. Der forventes derfor ikke at opstå bivirkninger eller negative hændelser som følge af interventionerne i dette studium. Der ses således ingen ulemper ved interventionerne. Skulle der imidlertid opstå bivirkninger eller negative hændelser som følge af terapien, vil det blive registreret og indberettet til Den Videnskabsetiske Komite. Se den engelske forsøgsprotokol for definition af bivirkninger/negative hændelser.

15.8. Databehandling

Projektet anmeldes til Datatilsynet. De forsøgsansvarlige vil indtaste patientens data direkte i en elektronisk CRF (Case Report Form) ved brug af dataindtastningssystemet REDCap. REDCap er et elektronisk dataindtastningssystem, der hostes af CIMT i Region Hovedstaden. REDCap er i overensstemmelse med den danske lovgivning for opbevaring af persondata (Datatilsynet). Data for hver patient er forbundet med et unikt løbenummer. Forsøgsansvarlige og forskningsassistenter er de eneste, der kan tilgå data i REDCap.

Projektet vil blive udført ved overholdelse af databeskyttelsesforordningen og databeskyttelsesloven.

Journaloplysninger

I forbindelse med henvisning til projektet fra teams i Region Hovedstadens Psykiatri eller Region Sjælland, kan det være nødvendigt at få videregivet oplysninger fra patientens journal med henblik på at identificere om patienten kan indgå i projektet (dvs. opfylder inklusionskriterierne og ikke opfylder eksklusionskriterier). De specifikke helbredsforhold, der indhentes oplysninger om fra journalen, er primært symptomniveau (f.eks. omfang af psykosesymptomer/negative symptomer, misbrug af stoffer, medicinsk behandling, begavelsesniveau) samt selvmordsrisiko. Formålet med dette er at afklare, om patienten opfylder inklusionskriterierne for projektinklusion (dvs. har psykoselidelse med niveau af negative symptomer svarende til ≥3 på SANS, ikke har misbrugsadfærd og ikke har intellektuel forringelse eller er svært suicidaltruet). Derudover indsamles data om medicin og ændring i dette med henblik på i de statistiske analyser at kunne udelukke, at en mulig behandlingseffekt kan tilskrives ændring i den psykofarmakologiske behandling. Patienterne forventes at være grundigt informeret om projektet, når de henvises til det, hvorfor det vurderes, at størstedelen af patienterne vil være interesseret i at blive inkluderet. For dog at give et konservativt estimat vurderes det, at ud af 50 adspurgte vil 20 takke nej til projektdeltagelse på dette tidspunkt. Derfor estimeres det, at det vil dreje sig om 50 journaler. Tidsperioden for journaloplysninger er fra patientens initiale kontakt til det psykiatriske system, da der skal indhentes information om evt. organisk hjernelidelses-diagnose og diagnose med lavt begavelsesniveau. Forsøgspersonens samtykke giver den forsøgsansvarlige, sponsor/sponsor repræsentant samt evt. kontrolmyndighed direkte adgang til at indhente relevante oplysninger i patientens journal med henblik på at se oplysninger om forsøgspersonens helbredsforhold, som er nødvendige som led i gennemførelse af forskningsprojekt samt i kontroløjemed, herunder egenkontrol, kvalitetskontrol og monitorering. Alle oplysninger som er indsamlet i projektet og fra journalen vil blive anvendt i projektet i anonymiseret form.

Håndtering af selvmordstanker/planer

Projektet følger god klinisk praksis for monitorering af selvmordsrisiko; herunder nyopståede eller forværring i selvmordstanker. Dette vurderes ved alle kliniske møder i projektet. Såfremt en patient oplever klinisk forværring og. eks. giver udtryk for nyopståede eller forværring i selvmordstanker, så er handleplanen at projektdeltagere, jf. det samtykke som er givet ved inklusion, straks tager kontakt til patientens behandlingsansvarlige kontaktperson i psykiatrien med henblik at vurdere behovet for at iværksætte relevante interventionstiltag (f.eks. intensivering af den ambulante behandling eller indlæggelse). Såfremt patienten ikke har et aktuelt forløb i psykiatrien, vil der blive taget kontakt til egen læge. Derudover vil det blive vurderet, om selvmordstankerne/evt. planer har en sådan karakter, at vedkommende skal følges til en vurdering og evt. indlæggelse i den psykiatriske modtagelse. Det vil også blive vurderet, om man fra projektets side skal iværksætte henvisning til ambulant psykiatrisk behandling. Derudover vil der blive taget stilling til, om patienten på baggrund af forværringen må ekskluderes fra forsøget. Deltagelse i projektet er altid frivilligt, og samtykke kan trækkes tilbage uden at dette har nogen form for indflydelse for patientens rettigheder eller behandling.

Erstatningsordning

Forsøget er omfattet af patienterstatningen.

15.9. Videnskabsetisk redegørelse

Projektet vil blive anmeldt til Videnskabsetisk Komité og Videnscenter for Datasikkerhed.

Patienter som indgår i projektet, vil blive grundigt informeret både mundtligt og skriftligt, og deltagelse i forsøget accepteres først efter informeret skriftligt samtykke er afgivet. Det vil blive understreget overfor patienterne, at de til enhver tid og uden begrundelse kan trække sig fra projektet, og at dette ikke vil påvirke deres fremtidige behandling i sundhedssystemet.

15.10. Fordelene for patienterne

Patienterne vil gennemgå et udredningsprogram samt have tæt personlig kontakt til de forsøgsansvarlige. Yderligere vil alle patienter modtage en målrettet intervention, der sigter mod at reducere negative symptomer ved psykose – et symptomkompleks man aktuelt ikke har en effektiv og målrettet behandling til.

Behandlingen og eventuelle negative hændelser vil blive fulgt tæt. Behandlingen er planlagt således, at den følger gældende kliniske retningslinjer.

Patienterne vil få mulighed for at få tilbagemelding på de undersøgelser, de gennemgår, hvorfor det vil give patienterne, og deres behandlere, yderligere indsigt i patientens individuelle symptomer og vanskeligheder.

15.11. Ulemperne for patienter

Der vurderes primært at være en ulempe forbundet med forsøget i form af muligheden for at blive ”køresyg” ved brug af virtual reality headset. Dette er dog ikke en hyppig bivirkning, og den kan som oftest afsvækkes ved at tilbyde patienten en langsommere indføring i virtual reality scenarierne. Yderligere kan der være en ulempe forbundet med forsøget i form af tidsforbruget ved undersøgelserne (der foretages ved baseline samt 3- måneders opfølgning). Tidsforbruget udgør dog kun ca. 3-4 timer undersøgelse pr. undersøgelse.

15.12. Studiets potentiale

Som angivet i engelsk forsøgsprotokol, har negative symptomer ved psykose stor indflydelse på patienternes kliniske og funktionelle prognose, men der findes aktuelt ikke nogen målrettet og evidensbaseret behandling til dette symptomkompleks. Der er således et åbenlyst behov for at finde effektive, engagerende og tolerable terapiformer til denne patientgruppe. Dette studium afprøver effekten af en korterevarende, manualiseret, virtual reality-baseret terapiform i et klinisk forsøg. Hvis VR-behandlingen viser sig effektiv, kan det skabe udgangspunkt for at afprøve den i et stort randomiseret klinisk forsøg mhp at skabe evidens og på sigt mulig implementering i relevante behandlingsenheder under tæt optræning og supervision.

15.13. Økonomiske forhold og klausuler

Forsøget har modtaget støtte i form af bevilling på 2.005.811 kr. fra Danmarks Frie Forskningsfond, der udbetales til forsøgsansvarligs forskningsafdeling.

Beløbet skal bruges til aflønning af en forskningsassistent i projektet samt psykolog, der udfører VR-behandlingen samt MR-skanninger og licens til digital assessment app.

Virtual reality-programmet er udviklet af den CleVR, en Hollandsk privatejet virksomhed, der er specialiseret i VR-løsninger. CleVR har ingen indflydelse på studiets design, dataindsamling, analyser eller præsentation af data, ligesom CleVR heller ikke har adgang til projektets data. CleVR har således ikke habilitetsproblemer i forhold til studiet. Forsøgsansvarlig har ingen økonomisk interesse til CleVR.

15.14. Initiativtager

Forskningsleder, specialpsykolog, dr.med, ph.d., lektor Louise Birkedal Glenthøj fra VIRTU Research Group på Psykiatrisk Center København har taget initiativ til at iværksætte projektet. VIRTU er i forvejen særdeles aktiv i forskning i virtual reality behandling i psykiatrien.

15.15. Vederlag

Patienter vil ikke modtage nogen godtgørelse, idet de er under behandling. De vil få godtgjort deres transportudgifter og få forplejning på undersøgelsesdagene.

15.16. Offentliggørelse

Såvel positive som negative og inkonklusive forskningsresultater vil blive offentliggjort i internationale tidsskrifter. Resultaterne vil ligeledes blive præsenteret på nationale og internationale møder og kongresser.

Projektet vil blive registreret på www.clinicaltrials.gov,når der foreligger godkendelse fra Videnskabsetisk Komité samt Videnscenter for Dataanmeldelser, og inden første patient er inkluderet i projektet.

15.17. Organisation

Psykiatrisk Center København stiller kontorlokaler til rådighed. Her vil interviews omkring symptomer-funktionsniveau foregå og blive udført af forsøgsansvarlige psykologer og læger. Hovedansvaret for behandlingen vil blive varetage af psykolog med specialviden inden for feltet. Projektgruppen består af forskningsleder og lektor PhD, dr.med. Louise Birkedal Glenthøj, professor Bjørn Ebdrup, Seniorforsker Tina Dam Kristensen, psykolog, specialist i psykoterapi Lise Mariegaard og forskningsassistent, psykolog Valentin Donath.

15.18. Gennemførlighed

Forskergruppen har stor erfaring med interventionsforsøg til patienter med psykiatriske lidelser og har nyligt afsluttet verdens største randomiserede kliniske forsøg til undersøgelse af VR-behandling til hørehallucinationer og paranoia. Der er er et godt og tæt samarbejde med kliniske forskningsenheder i Region Hovedstadens Psykiatri, der gerne henviser patienter til projektet. Baseret på, at vi netop har inkluderet 516 personer med psykose i lignende forskningsprojekter, synes det realistisk at inkludere 30 personer i dette projekt.

16. References

Abram, S. V., Wisner, K. M., Fox, J. M., Barch, D. M., Wang, L., Csernansky, J. G., MacDonald, A. W., & Smith, M. J. (2017). Fronto-temporal connectivity predicts cognitive empathy deficits and experiential negative symptoms in schizophrenia. *Human Brain Mapping*, *38*(3), 1111–1124. https://doi.org/10.1002/hbm.23439

Addington, D., Addington, J., & Maticka-Tyndale, E. (1993). Assessing depression in schizophrenia: the Calgary Depression Scale. *The British Journal of Psychiatry. Supplement*, *22*, 39–44.

Albert, N., Bertelsen, M., Thorup, A., Petersen, L., Jeppesen, P., Le Quack, P., Krarup, G., Jørgensen, P., & Nordentoft, M. (2011). Predictors of recovery from psychosis Analyses of clinical and social factors associated with recovery among patients with first-episode psychosis after 5 years. *Schizophrenia Research*, *125*(2–3), 257–266. https://doi.org/10.1016/j.schres.2010.10.013

Albert, N., Melau, M., Jensen, H., Emborg, C., Jepsen, J. R. M., Fagerlund, B., Gluud, C., Mors, O., Hjorthøj, C., & Nordentoft, M. (2017). Five years of specialised early intervention versus two years of specialised early intervention followed by three years of standard treatment for patients with a first episode psychosis: randomised, superiority, parallel group trial in Denmark (OPUS II). *BMJ (Clinical Research Ed.)*, *356*, i6681.

Andreasen, N. C. (1984). Scale for assesment of negative symptomes/Scale for assesment of positive symptomes. *University of Iowa Press*.

Attkisson, C. C., & Zwick, R. (1982). The client satisfaction questionnaire. *Evaluation and Program Planning*, *5*(3), 233–237. https://doi.org/10.1016/0149-7189(82)90074-X

Austin, S. F., Mors, O., Budtz-Jørgensen, E., Secher, R. G., Hjorthøj, C. R., Bertelsen, M., Jeppesen, P., Petersen, L., Thorup, A., & Nordentoft, M. (2015). Long-term trajectories of positive and negative symptoms in first episode psychosis: A 10year follow-up study in the OPUS cohort. *Schizophrenia Research*, *168*(1–2), 84–91. https://doi.org/10.1016/j.schres.2015.07.021

Bègue, I., Kaiser, S., & Kirschner, M. (2020). Pathophysiology of negative symptom dimensions of schizophrenia – Current developments and implications for treatment. *Neuroscience & Biobehavioral Reviews*, *116*, 74–88. https://doi.org/10.1016/j.neubiorev.2020.06.004

Bell, M. L., Whitehead, A. L., & Julious, S. A. (2018). Guidance for using pilot studies to inform the design of intervention trials with continuous outcomes. *Clinical Epidemiology*, *Volume 10*, 153–157. https://doi.org/10.2147/CLEP.S146397

Birchwood, M., Smith, J., Cochrane, R., Wetton, S., & Copestake, S. (1990). The Social Functioning Scale. The development and validation of a new scale of social adjustment for use in family intervention programmes with schizophrenic patients. *British Journal of Psychiatry*, *157*(DEC.), 853–859. https://doi.org/10.1192/bjp.157.6.853

Browne, J., Harvey, P. D., Buchanan, R. W., Kelly, D. L., Strauss, G. P., Gold, J. M., Holden, J. L., & Granholm, E. (2022). A Longitudinal Examination of Real-World Sedentary Behavior in Adults with Schizophrenia-Spectrum Disorders in a Clinical Trial of Combined Oxytocin and Cognitive Behavioral Social Skills Training. *Behavioral Sciences*, *12*(3). https://doi.org/10.3390/bs12030060

Cella, M., Bishara, A. J., Medin, E., Swan, S., Reeder, C., & Wykes, T. (2014). Identifying cognitive remediation change through computational modelling - Effects on reinforcement learning in schizophrenia. *Schizophrenia Bulletin*, *40*(6), 1422–1432. https://doi.org/10.1093/schbul/sbt152

Correll, C. U., & Schooler, N. R. (2020). Negative symptoms in schizophrenia: A review and clinical guide for recognition, assessment, and treatment. *Neuropsychiatric Disease and Treatment*, *16*, 519–534. https://doi.org/10.2147/NDT.S225643

Devoe, D. J., Lu, L., Cannon, T. D., Cadenhead, K. S., Cornblatt, B. A., McGlashan, T. H., Perkins, D. O., Seidman, L. J., Tsuang, M. T., Woods, S. W., Walker, E. F., Mathalon, D. H., Bearden, C. E., & Addington, J. (2020). Persistent negative symptoms in youth at clinical high risk for psychosis: A longitudinal study. *Schizophrenia Research*, *xxxx*. https://doi.org/10.1016/j.schres.2020.04.004

Dollfus, S., Mach, C., & Morello, R. (2016). Self-Evaluation of Negative Symptoms: A Novel Tool to Assess Negative Symptoms. *Schizophrenia Bulletin*, *42*(3), 571–578. https://doi.org/10.1093/schbul/sbv161

Eack, S. M., & Newhill, C. E. (2007). Psychiatric symptoms and quality of life in schizophrenia: A meta-analysis. *Schizophrenia Bulletin*, *33*(5), 1225–1237. https://doi.org/10.1093/schbul/sbl071

Forlim, C. G., Klock, L., Bächle, J., Stoll, L., Giemsa, P., Fuchs, M., Schoofs, N., Montag, C., Gallinat, J., & Kühn, S. (2020). Reduced Resting-State Connectivity in the Precuneus is correlated with Apathy in Patients with Schizophrenia. *Scientific Reports*, *10*(1), 2616. https://doi.org/10.1038/s41598-020-59393-6

Freeman, D., Reeve, S., Robinson, A., Ehlers, A., Clark, D., Spanlang, B., & Slater, M. (2017). Virtual reality in the assessment, understanding, and treatment of mental health disorders. *Psychological Medicine*, *47*(14), 2393–2400. https://doi.org/10.1017/S003329171700040X

Fusar-Poli, P., Papanastasiou, E., Stahl, D., Rocchetti, M., Carpenter, W., Shergill, S., & McGuire, P. (2015). Treatments of Negative Symptoms in Schizophrenia: Meta-Analysis of 168 Randomized Placebo-Controlled Trials. *Schizophrenia Bulletin*, *41*(4), 892–899. https://doi.org/10.1093/schbul/sbu170

Galderisi, S., Kaiser, S., Bitter, I., Nordentoft, M., Mucci, A., Sabé, M., Giordano, G. M., Nielsen, M. Ø., Glenthøj, L. B., Pezzella, P., Falkai, P., Dollfus, S., & Gaebel, W. (2021). EPA guidance on treatment of negative symptoms in schizophrenia. *European Psychiatry*, *64*(1). https://doi.org/10.1192/j.eurpsy.2021.13

Ghomi, M., Wrightman, M., Ghaemian, A., Grey, N., Pickup, T., & Richardson, T. (2021). Development and validation of the Readiness for Therapy Questionnaire (RTQ). *Behavioural and Cognitive Psychotherapy*, *49*(4), 413–425. https://doi.org/10.1017/S1352465820000764

Giordano, G. M., Caporusso, E., Pezzella, P., & Galderisi, S. (2022). Updated perspectives on the clinical significance of negative symptoms in patients with schizophrenia. *Expert Review of Neurotherapeutics*, *22*(7), 541–555. https://doi.org/10.1080/14737175.2022.2092402

Global Burden of Disease Study 2013 Collaborators. (2015). Global, regional, and national incidence, prevalence, and years lived with disability for 301 acute and chronic diseases and injuries in 188 countries, 1990-2013: A systematic analysis for the Global Burden of Disease Study 2013. *The Lancet*, *386*(9995), 743–800. https://doi.org/10.1016/S0140-6736(15)60692-4

Gold, J. M., Waltz, J. A., Prentice, K. J., Morris, S. E., & Heerey, E. A. (2008). Reward processing in schizophrenia: A deficit in the representation of value. *Schizophrenia Bulletin*, *34*(5), 835–847. https://doi.org/10.1093/schbul/sbn068

Gooding, D. C., & Pflum, M. J. (2014). The assessment of interpersonal pleasure: Introduction of the Anticipatory and Consummatory Interpersonal Pleasure Scale (ACIPS) and preliminary findings. *Psychiatry Research*, *215*(1), 237–243. https://doi.org/10.1016/j.psychres.2013.10.012

Grant, P. M., & Beck, A. T. (2009). Defeatist beliefs as a mediator of cognitive impairment, negative symptoms, and functioning in schizophrenia. *Schizophrenia Bulletin*, *35*(4), 798–806. https://doi.org/10.1093/schbul/sbn008

Grill, F., Nyberg, L., & Rieckmann, A. (2021). Neural correlates of reward processing: Functional dissociation of two components within the ventral striatum. *Brain and Behavior*, *11*(2), 1–12. https://doi.org/10.1002/brb3.1987

Hakulinen, C., McGrath, J. J., Timmerman, A., Skipper, N., Mortensen, P. B., Pedersen, C. B., & Agerbo, E. (2019). The association between early-onset schizophrenia with employment, income, education, and cohabitation status: nationwide study with 35 years of follow-up. *Social Psychiatry and Psychiatric Epidemiology*, *54*(11), 1343–1351. https://doi.org/10.1007/s00127-019-01756-0

Hjorthøj, C., Stürup, A. E., Mcgrath, J. J., & Nordentoft, M. (2017). Years of potential life lost and life expectancy in schizophrenia : a systematic review and meta-analysis. *Lancet Psychiatry*, *0366*(published online Feb 21), 1–7. https://doi.org/10.1016/S2215-0366(17)30078-0

Jeppesen, U. N., Due, A. S., Mariegaard, L., Pinkham, A., Vos, M., Veling, W., Nordentoft, M., & Glenthøj, L. B. (2022). Face Your Fears: Virtual reality-based cognitive behavioral therapy (VR-CBT) versus standard CBT for paranoid ideations in patients with schizophrenia spectrum disorders: a randomized clinical trial. *Trials*, *23*(1), 658. https://doi.org/10.1186/s13063-022-06614-0

Kennedy, R. S., Fowlkes, J. E., Berbaum, K. S., & Lilienthal, M. G. (1992). Use of a motion sickness history questionnaire for prediction of simulator sickness. *Aviation, Space, and Environmental Medicine*, *63*(7), 588–593.

Kirkpatrick, B., Strauss, G. P., Nguyen, L., Fischer, B. A., Daniel, D. G., Cienfuegos, A., & Marder, S. R. (2011). The brief negative symptom scale: Psychometric properties. *Schizophrenia Bulletin*, *37*(2), 300–305. https://doi.org/10.1093/schbul/sbq059

Morosini, P. L., Magliano, L., Brambilla, L., Ugolini, S., & Pioli, R. (2000). Development, reliability and acceptability of a new version of the DSM-IV Social and Occupational Functioning Assessment Scale (SOFAS) to assess routine social functioning. *Acta Psychiatrica Scandinavica*, *101*(4), 323–329. https://doi.org/10.1034/j.1600-0447.2000.101004323.x

Newnham, E. A., Hooke, G. R., & Page, A. C. (2010). Monitoring treatment response and outcomes using the World Health Organization's Wellbeing Index in psychiatric care. *Journal of Affective Disorders*, *122*(1–2), 133–138. https://doi.org/10.1016/j.jad.2009.06.005

Nielsen, M. Ø., Rostrup, E., Broberg, B. V., Wulff, S., & Glenthøj, B. (2018). Negative Symptoms and Reward Disturbances in Schizophrenia Before and After Antipsychotic Monotherapy. *Clinical EEG and Neuroscience*, *49*(1), 36–45. https://doi.org/10.1177/1550059417744120

Novo, A., Fonsêca, J., Barroso, B., Guimarães, M., Louro, A., Fernandes, H., Lopes, R. P., & Leitão, P. (2021). Virtual reality rehabilitation's impact on negative symptoms and psychosocial rehabilitation in schizophrenia spectrum disorder: A systematic review. *Healthcare (Switzerland)*, *9*(11), 1–11. https://doi.org/10.3390/healthcare9111429

Pot-kolder, R. M. C. A., Geraets, C. N. W., Veling, W., Beilen, M. Van, Staring, A. B. P., Gijsman, H. J., & Delespaul, P. A. E. G. (2018). *Articles Virtual-reality-based cognitive behavioural therapy versus waiting list control for paranoid ideation and social avoidance in patients with psychotic disorders : a single-blind randomised controlled trial*. *0366*(18).

Rus-Calafell, M., Garety, P., Sason, E., Craig, T. J. K. K., & Valmaggia, L. R. (2018). Virtual reality in the assessment and treatment of psychosis: a systematic review of its utility, acceptability and effectiveness. *Psychological Medicine*, *48*(3), 362–391. https://doi.org/10.1017/S0033291717001945

Sahakian, B. J., & Owen, a M. (1992). Computerized assessment in neuropsychiatry using CANTAB: discussion paper. *The Royal Society of Medicine*, *85*(July), 399–402.

Smith, L. C., Mariegaard, L., Vernal, D. L., Christensen, A. G., Albert, N., Thomas, N., Hjorthøj, C., Glenthøj, L. B., & Nordentoft, M. (2022). The CHALLENGE trial: the effects of a virtual reality-assisted exposure therapy for persistent auditory hallucinations versus supportive counselling in people with psychosis: study protocol for a randomised clinical trial. *Trials*, *23*(1), 773. https://doi.org/10.1186/s13063-022-06683-1

Spreckelmeyer, K. N., Krach, S., Kohls, G., Rademacher, L., Irmak, A., Konrad, K., Kircher, T., & Gründer, G. (2009). Anticipation of monetary and social reward differently activates mesolimbic brain structures in men and women. *Social Cognitive and Affective Neuroscience*, *4*(2), 158–165. https://doi.org/10.1093/scan/nsn051

Strauss, G. P., Waltz, J. A., & Gold, J. M. (2014). A review of reward processing and motivational impairment in schizophrenia. *Schizophrenia Bulletin*, *40*(SUPPL. 2), 107–116. https://doi.org/10.1093/schbul/sbt197

Van Der Meer, L., Kaiser, S., & Castelein, S. (2021). Negative symptoms in schizophrenia: Reconsidering evidence and focus in clinical trials. *British Journal of Psychiatry*, *219*(1), 359–360. https://doi.org/10.1192/bjp.2021.66

Ventura, J., Hellemann, G. S., Thames, A. D., Koellner, V., & Nuechterlein, K. H. (2009). Symptoms as mediators of the relationship between neurocognition and functional outcome in schizophrenia: a meta-analysis. *Schizophrenia Research*, *113*(2–3), 189–199. https://doi.org/10.1016/j.schres.2009.03.035

Wang, K. S., Smith, D. V., & Delgado, M. R. (2016). Using fMRI to study reward processing in humans: past, present, and future. *Journal of Neurophysiology*, *115*(3), 1664–1678. https://doi.org/10.1152/jn.00333.2015

Whitton, A. E., Treadway, M. T., & Pizzagalli, D. A. (2015). Reward processing dysfunction in major depression, bipolar disorder and schizophrenia. *Current Opinion in Psychiatry*, *28*(1), 7–12. https://doi.org/10.1097/YCO.0000000000000122

Witmer BG, S. M. (1998). Measuring Presence in Virtual Environments: A Presence Questionnaire. *Presence: Teleoperators and Virutal Environments*, *7*, 225–240.

Zald, D. H., & Treadway, M. T. (2017). Reward Processing, Neuroeconomics, and Psychopathology. *Annual Review of Clinical Psychology*, *13*, 471–495. https://doi.org/10.1146/annurev-clinpsy-032816-044957

15. Danish addendum to study protocol

15.1. Informed consent and recruitment

Recruitment of patients will be done in collaboration with healthcare professionals from the psychiatric units in the Capital Region of Denmark or in Region Zealand. The staff of the outpatient treatment units will mediate contact with the project managers if the patient agrees to this.

The project will also be advertised on the Capital Region of Denmark's own site, Research Portal for Patient Inclusion. This platform is specifically designed to present ongoing research projects to patients and their relatives who might be interested in participating in such studies.

Regardless of recruitment method, it is a prerequisite that the patient meets the criteria for participation in the project and that the patient's primary care provider is informed before the project starts.

Written information about the project will be sent/provided at least 48 hours before the first visit to VIRTU Research Group, Psychiatric Center Copenhagen, and the patient will be informed about the right to bring an advisor.

During the process, the investigators will be in contact with the patient's contact person in e.g. OPUS or district psychiatric center, if the patient agrees to this. It is a prerequisite that the patient fulfills the criteria for participation in the project before the project starts.

Upon arrival, the patient is informed orally about the project by the project's research assistant, who answers any questions before the patient signs the consent forms. The patient is offered a reflection period of 24 hours after receiving oral and written information about the project.
Patients who express a wish to do so can consent immediately. The leaflet "Subjects' rights in health research projects" is handed out together with the participant information.

The interview will take place in a closed room, possibly with the patient's counselor. In the event of questions or complications, the patient will have the opportunity to contact the study coordinators or other healthcare professionals associated with VIRTU Research Group, Psychiatric Center Copenhagen by telephone throughout the trial period.

During the course of the study, the study coordinators will be in contact with the patient's contact person in the psychiatric treatment program, if the patient consents to this.

15.2. Frequency of contact

At baseline and 3 months after the end of treatment, assessment of psychopathology, functional level, MRI scans and side effects/negative events will be performed. If a subject discontinues the protocol, treatment will be offered according to relevant guidelines in psychiatry in the Capital Region of Denmark or Region Zealand.

Criteria for discontinuation

Study participants can leave the study at any time, participation is voluntary. Herewith specified reasons for discontinuation of the study:

i. Voluntary withdrawal by the patient; the patient can withdraw from the study at any time without affecting their further treatment in the healthcare system.
ii. Incorrect inclusion of the patient (e.g. the patient does not meet the inclusion/exclusion criteria).

Procedure for discontinuation

Patients who discontinue the intervention during hospitalization are fully entitled to do so (see voluntary principle). Patients who wish to withdraw from the intervention will be offered the opportunity to answer a questionnaire about their satisfaction with the treatment if they so wish.

15.3. Surveys in the study

Symptom and functional level

As stated in the protocol, assessments to evaluate patients' symptom level and level of function are performed using interviews and self-report forms. In addition, more qualitative questions are asked of the participants. The qualitative questions concern the participants' experience of receiving therapy using virtual reality and what they felt was particularly effective or could be improved.

Free speech interview

Participants will undergo a free speech interview with a research assistant lasting 15-20 minutes. This is audio-recorded and includes questions about events in the participant's life and their mental health.

15.4. Digital assessment

Participants will also receive questions on their cell phone about their symptom level and level of function. This is done via the Monsenso app. This can be downloaded to the participant's own phone or a smartphone can be given to the participants during the project period, depending on the participant's preference. The app also records geolocation and acceleration data that will be used to map the users' activity level.

The purpose of the digital assessment is to get a more ecologically valid measure of fluctuations in symptom and functional level and thereby be able to describe possible changes as a result of the intervention in more detail. In the week leading up to the start of any intervention and the week immediately following the end of the intervention

questions are sent five times a day via the app with the aim of getting a detailed picture of the participants' everyday function and symptom level. During the remaining 12-week project period, questions are sent once a day (evening).

15.5. MRI scans

The scans are described in the protocol above. MRI scans provide the opportunity to obtain structural and functional data on brain function. The scans are performed by trained staff at Glostrup Hospital and last a maximum of 90 minutes including a break. Professor and Consultant Bjørn Ebdrup will be clinically responsible for MRI scans.

15.6. Time consumption

In total, the surveys take about 4 hours to complete at baseline and about 3 hours at 3-month follow-up.

15.7. Treatment modalities

The manualized virtual reality intervention is described in the UK trial protocol.

Medication

No medication is offered as part of this study. Any psychopharmacological treatment will be handled by the patient's primary care provider in their psychiatric program. If a project participant is on medication and, upon contact with the project, reports side effects, the research project will contact the patient's primary care provider.

Side effects, risks and disadvantages of the trial

There may be side effects from the trial in the form of discomfort from using virtual reality glasses and headphones and possibly feeling dizzy or motion sickness, but generally no side effects are reported for virtual reality treatment.[(40)]The form of therapy tested is comparable to VR therapy, which we have tested in another trial to reduce paranoia (Jeppesen et al, 2022), and which has also been tested internationally (Pot-kolder et al., 2018), has only shown beneficial effects in reducing patients' symptom levels and their associated distress and discomfort. Therefore, no side effects or adverse events are expected to occur as a result of the interventions in this study. Thus, no disadvantages are seen with the interventions. However, should side effects or adverse events occur as a result of the therapy, they will be recorded and reported to the Research Ethics Committee. See the English trial protocol for the definition of side effects/negative events.

15.8. Data processing

The project is notified to the Danish Data Protection Agency. The investigators will enter patient data directly into an electronic CRF (Case Report Form) using the data entry system REDCap. REDCap is an electronic data entry system hosted by CIMT in the Capital Region of Denmark. REDCap complies with the Danish legislation for storage of personal data (Datatilsynet). Data for each patient is associated with a unique serial number. The principal investigators and research assistants are the only ones who can access data in REDCap.

The project will be conducted in compliance with the General Data Protection Regulation and the Data Protection Act.

Journal information

In connection with referrals to the project from teams in the Capital Region of Denmark's Psychiatry or Region Zealand, it may be necessary to obtain information from the patient's medical record in order to identify whether the patient can be included in the project (i.e. meets the inclusion criteria and does not meet the exclusion criteria). The specific health conditions that are collected from the medical record are primarily symptom level (e.g. extent of psychosis symptoms/negative symptoms, substance abuse, medical treatment, intelligence level) and suicide risk. The purpose of this is to determine if the patient meets the inclusion criteria for project inclusion (i.e. has a psychosis disorder with a level of negative symptoms corresponding to ≥3 on the SANS, does not have substance abuse behavior and does not have intellectual impairment or is severely suicidal). In addition, data on medication and changes in medication will be collected in order to exclude in the statistical analyses that a possible treatment effect can be attributed to a change in psychopharmacological treatment. Patients are expected to be thoroughly informed about the project when they are referred to it, which is why it is estimated that the majority of patients will be interested in being included. However, to give a conservative estimate, it is estimated that out of 50 respondents, 20 will decline to participate in the project at this stage. Therefore, it is estimated that it will be about 50 records.

The time period for medical record information is from the patient's initial contact with the psychiatric system, as information on any organic brain disorder diagnosis and diagnosis of mental retardation must be obtained.

The subject's consent gives the investigator, sponsor/sponsor representative and any regulatory authority direct access to obtain relevant information in the patient's medical record in order to view information about the subject's health conditions that is necessary for the conduct of the research project and for control purposes, including self-monitoring, quality control and monitoring. All information collected in the project and from the medical record will be used in the project in anonymized form.

Management of suicidal thoughts/plans

The project follows good clinical practice for monitoring suicide risk; including new or worsening suicidal thoughts. This is assessed at all clinical meetings in the project. If a patient experiences clinical deterioration and, for example, expresses new or worsening suicidal ideation, the action plan is that project participants, cf. the consent given at inclusion, immediately contact the patient's responsible contact person in psychiatry to assess the need to initiate relevant interventions (e.g. intensification of outpatient treatment or hospitalization). If the patient does not have a current course of treatment in psychiatry, contact will be made with their own doctor. In addition, it will be assessed whether the suicidal thoughts/possible plans are of such a nature that the patient should be followed for an assessment and possible hospitalization in the psychiatric ward. It will also be assessed whether the project should initiate referral for outpatient psychiatric treatment. In addition, it will be decided whether the patient should be excluded from the trial based on the deterioration. Participation in the project is always voluntary, and consent can be withdrawn without affecting the patient's rights or treatment in any way.

Compensation scheme

The trial is covered by patient compensation.

15.9. Research ethics statement

The project will be notified to the Research Ethics Committee and the Danish Data Protection Agency.

Patients participating in the project will be thoroughly informed both orally and in writing, and participation in the trial will only be accepted after informed written consent has been given. It will be emphasized to patients that they can withdraw from the study at any time and without reason and that this will not affect their future treatment in the healthcare system.

15.10. The benefits for patients

Patients will undergo an assessment program and have close personal contact with the investigators. Furthermore, all patients will receive a targeted intervention aimed at reducing negative symptoms of psychosis - a symptom complex for which there is currently no effective and targeted treatment.

The treatment and any adverse events will be closely monitored. The treatment is planned to follow current clinical guidelines.

Patients will have the opportunity to receive feedback on the examinations they undergo, which will give patients and their caregivers further insight into the patient's individual symptoms and difficulties.

15.11. The disadvantages for patients

The main disadvantage associated with the trial is considered to be the possibility of "motion sickness" when using the virtual reality headset. However, this is not a frequent side effect and can usually be mitigated by offering the patient a slower introduction to the virtual reality scenarios. There may also be a disadvantage associated with the trial in terms of the time required for the examinations (performed at baseline and 3-month follow-up). However, the time spent is only about 3-4 hours of study per study.

15.12. The potential of the study

As indicated in the UK trial protocol, negative symptoms in psychosis have a major impact on patients' clinical and functional prognosis, but there is currently no targeted and evidence-based treatment for this symptom complex. Thus, there is an obvious need to find effective, engaging and tolerable therapies for this patient group. This study tests the efficacy of a short-term, manualized, virtual reality-based therapy in a clinical trial. If the VR therapy proves to be effective, it may create a basis for testing it in a large randomized clinical trial in order to create evidence and eventually possible implementation in relevant treatment units under close training and supervision.

15.13. Financial conditions and clauses

The trial has received funding in the form of a grant of DKK 2,005,811 from the Independent Research Fund Denmark, which is paid to the principal investigator's research department.

The amount will be used for the salary of a research assistant in the project as well as a psychologist who performs the VR treatment as well as MRI scans and license for digital assessment app.

The virtual reality program is developed by CleVR, a Dutch privately owned company that specializes in VR solutions. CleVR has no influence on the study's design, data collection, analysis or presentation of data, nor does CleVR have access to the project data. CleVR thus has no impartiality issues in relation to the study. The investigator has no financial interest in CleVR.

15.14. Initiator of the study

Head of research, specialist psychologist, PhD, Associate Professor Louise Birkedal Glenthøj from VIRTU Research Group at Psychiatric Center Copenhagen has taken the initiative to launch the project. VIRTU is already very active in research in virtual reality treatment in psychiatry.

15.15. Remuneration

Patients will not receive any compensation as they are undergoing treatment. They will be reimbursed for their transportation costs and will be provided with meals on the study days.

15.16. Disclosure

Positive, negative and inconclusive research results will be published in international journals. Results will also be presented at national and international meetings and congresses.

The project will be registered at www.clinicaltrials.gov,når with approval from the Research Ethics Committee and the Knowledge Center for Data Reviews, and before the first patient is included in the project.

15.17. Organization

Psychiatric Center Copenhagen provides office space. Here, interviews about symptom-function level will take place and be conducted by the psychologists and doctors responsible for the study. The main responsibility for the treatment will be handled by a psychologist with specialized knowledge in the field. The project group consists of research leader and associate professor PhD, dr.med. Louise Birkedal Glenthøj, Professor Bjørn Ebdrup, Senior Researcher Tina Dam Kristensen, psychologist, specialist in psychotherapy Lise Mariegaard and research assistant, psychologist Valentin Donath.

15.18. Feasibility

The research group has extensive experience with intervention trials for patients with psychiatric disorders and has recently completed the world's largest randomized clinical trial investigating VR treatment for auditory hallucinations and paranoia. There is a good and close collaboration with clinical research units in the Capital Region of Denmark's Psychiatry, who are happy to refer patients to the project. Based on the fact that we have just included 516 people with psychosis in similar research projects, it seems realistic to include 30 people in this project.
